# The effects of sexual shame, emotion regulation and gender on sexual desire

**DOI:** 10.1038/s41598-023-31181-y

**Published:** 2023-03-10

**Authors:** K. W. Sævik, C. Konijnenberg

**Affiliations:** Department of Psychology, Inland Norway University of Applied Sciences, Lillehammer, Norway

**Keywords:** Psychology, Human behaviour

## Abstract

Sexual desire is of importance to sexual health, functioning, and well-being. Although an increasing number of studies address disorders related to sexual functioning, there is still a limited understanding of the underlying individual factors affecting sexual desire. The aim of the current study was to investigate the effect of sexual shame, emotion regulation strategies, and gender on sexual desire. In order to investigate this, sexual desire, expressive suppression, cognitive reappraisal, and sexual shame was measured in 218 Norwegian participants using the Emotion Regulation Questionnaire-10, the Sexual Desire Inventory-2, and the Sexual Shame Index-Revised. A multiple regression analysis indicated that cognitive reappraisal predicted sexual desire, β = 0.343, (218) = 5.09, *p* < 001, CI [0.407, 0.920], whereas sexual shame and expressive suppression were unrelated to sexual desire. Men scored significantly higher than women on expressive suppression, F(1, 216) = 24.968, p < 0.001; partial η^2^ = 0.104. The current study did not find any significant differences between women and men on cognitive reappraisal, sexual desire or sexual shame, all p > 0.05. Results from the current study indicates that the inclination toward cognitive reappraisal as a preferred emotion regulation strategy may positively affect the strength of sexual desire.

## Introduction

Sexual function is an important predictor for quality of life and is affected by biological, psychological and sociocultural factors^1^. Although considerable research has focused on biological and psychological factors related to sexual functioning, research into sociocultural influences on sexual desire is limited^2^. Particularly female sexuality has long been stigmatized and ignored although it has slowly gained more attention in both the healthcare sector and academia^3^. In western society, there is a long history of expectations of modesty and passivity for women, and of sexual grandeur and performance for men^4^. It was long believed that men both think about sex significantly more frequently and score higher on sexual desire compared to women and that women experience higher ratings of sexual shame compared to men^4,5^. However, this set of expectations may no longer mirror the current situation. Baumeister^6^ found that men have a higher degree of sexual desire compared to women in 2001, but more recent studies showed that the gender gap is diminishing^4,7,8^.

### Sexual desire

Sexual desire can be triggered by internal or external factors and is often defined as the motivation to engage in sexual activity, although some have also argued that it can appear spontaneously^9,10^. At the biological level, sexual desire is often seen as preceding sexual arousal and sexual behavior, and as a modulator of the two. While some studies suggest that sexual desire, sexual arousal, and sexual behavior are highly associated, others have failed to observe such an association^7^. Psychologically, sexual desire is seen as a motivational state fundamental for relationship- and sexual satisfaction, although the way it manifests is subject to both inter- and intrapersonal factors^11^. On the social level, sexual desire is related to sociocultural norms and expected behavioral patterns that can be affected by gender stereotypes^12^. When comparing men and women, studies have found that women generally were more likely to endorse desire for intimacy, emotional closeness, love and feeling sexually desirable while men were more likely to endorse desire for sexual release, orgasm, and pleasing their partner^7^.

### Sexual shame

Shame as an umbrella-term for a set of emotions, is invoked both externally by others, and internally by oneself. It can manifest in feelings of helplessness, inadequacy, being defect, and feeling inferior^13^. Shame affects intrapersonal health, interpersonal relationships, and is related to negative psychological health outcomes, including depression, self-efficacy, and poor overall mental health^5,14,15^. Sexual shame is a specific type of shame that refers to a feeling of disgust or humiliation towards one’s own identity as a sexual being and is composed of three factors: (1) relationship sexual shame, (2) internalized sexual shame, and (3) sexual inferiority^13^. Relational sexual shame includes interactions with another person and highlights feelings involving others. Internalized shame involves feelings of humiliation, disgust, abnormality, and inferiority which can be exemplified by being ashamed by one’s own body. Sexual inferiority refers to feelings of not fulfilling the self-experienced expectancies, often derived from societal norms and expectations and culture^5^. Sexual shame has been found to be related to self-hostility, sexual and relational dysfunctions, body-shaming, aggression, hypersexuality and sexual addiction^16^. Through sexual shame, a higher level of self-consciousness is generated that can lead to a maladaptive trail of thoughts, attitudes, beliefs, and behaviors, such as reduced self-esteem^5,16,17^.

### Emotion regulation

Emotion regulation is the ability to evaluate and adapt emotional responses involving experiential, physiological, and behavioral systems^18^. It is influenced by cognitive reappraisal, which refers to the reinterpretation of a situation that triggers emotion, thereby altering the meaning and its emotional impact^19,20^. In addition, emotional expression can be suppressed in an attempt to hide or inhibit emotional behavior, called expressive suppression^21^. Research on emotion regulation strategies have found positive effects of cognitive reappraisal and negative effects of expressive suppression on health and wellbeing outcomes^20,21^. Men and women use different emotion regulation strategies, with women using cognitive reappraisal more often compared to men while men are more likely to use expressive suppression strategies^22,23^. In relation to sexual health, the ability to use emotion regulation strategies, such as downregulating negative emotions, is beneficial for sexual functioning^24^. For example, reappraisal of a negative sexual experience may be more beneficial than suppressing shameful emotions related to the event.

### Current study

The main aim of the current study is to investigate the influence of sexual shame, emotion regulation strategies and gender on sexual desire. Based on previous literature, it was predicted that expressive suppression and sexual shame would negatively affect sexual desire, while cognitive reappraisal would have a positive effect on sexual desire. Additionally, it was predicted that men would score higher on sexual desire and expressive suppression and lower on sexual shame and cognitive reappraisal, compared to women. To our awareness, no recent studies have investigated how emotion regulation strategies and sexual shame is related to sexual desire. Furthermore, in light of current events such as the #Me too movement, gender perceptions may be changing, including traditional views of masculine and feminine sexual behavior. It is therefore important to continue to develop our knowledge of the structures, processes, and factors involved in sexual desire as it plays an essential role in sexual-, mental-and physical well-being.

## Materials and methods

### Participants and procedure

A total of 222 participants from Norway participated in the study, which was conducted online. Four participants were excluded due to picking several answer-options on separate single-answer questions. Consequently, the final sample comprised of 218 participants (69% female). Participants aged 18 and above were eligible for the study. Most respondents fell in the age group 18–23 years (50.9%, N = 111), followed by ages 24–29 years (30.7%, N = 67), 30–39 (15.1%, N = 33), and 40 + (3.3%, N = 7). The relationship status of the participants was mostly ‘Single’ (41.3%, N = 90), followed by ‘In a relationship but not living with partner’ (28%, N = 61), ‘In a relationship living with partner’ (26.1%, N = 57), and ‘Married living with their partner’ (5%, N = 11). Assessing whether the participants were monogamous gave a total of 192 identifying as monogamous (88.2%), 20 who identified as non-monogamous (9.1%), and 8 who did not want to answer (3.6%). Participants were recruited using a snowball sampling technique. The survey was advertised using social media groups related to the university where the authors are affiliated. Participation was anonymous and voluntary and written informed consent was obtained from all participants. The study was approved by the local ethics committee at Inland Norway University of Applied Sciences (LEFK 21/01894) and conducted in accordance with the Declaration of Helsinki (World Medical Association, 2011)^25^.

### Measures

An online self-report questionnaire was administered containing demographic questions and three self-report questionnaires assessing emotion regulation, sexual desire, and sexual shame.

#### *Sexual desire inventory-2 (SDI-2)*^26^

The SDI-2 is a modified version of the original SDI questionnaire designed to measure individual’s sexual desire. It consists of three dimensions: (1) partner-focused dyadic sexual desire (5 items), (2) general dyadic sexual desire for an attractive person (2 items), and (3) solitary sexual desire (4 items). An example question of partner-focused dyadic sexual desire is ‘During the last month, how often have you had sexual thoughts involving a partner?’, an example of general dyadic sexual desire for an attractive person is ‘When you first see an attractive person, how strong is your sexual desire?’, and an example of solitary sexual desire is ‘How strong is your desire to engage in sexual behavior by yourself?’. Seven items are scored on a 7-point Likert-scale while the other 4 items are scored on an 8-point Likert-scale. Scores were added to create a total score of sexual desire, where higher scores indicate higher levels of sexual desire (ranging from 0 to 101). The SDI-2 has been shown to have good psychometric properties with Cronbach alpha values ranging from 0.89 to 0.96. In addition, research has shown a strong concurrent validity between sexual desire scores on the SDI and sexual behavior^26^.

#### Emotion regulation questionnaire (ERQ-10)^18^

ERQ-10 is a 10-item self-report consisting of the subscales cognitive reappraisal (6 items) and expressive suppression (4 items). An example of a reappraisal item is ‘When I want to feel more positive emotion, I change the way I’m thinking about the situation’. An example of a suppression item is ‘I control my emotions by not expressing them’. The items were rated on a 7-point Likert-scale ranging from strongly disagree to strongly agree. Cronbach’s alpha has been found to range from 0.76 to 0.83 for the ERQ-10, indicating a good level of internal consistency^27^.

#### Sexual shame inventory—revised (SSI-R)^5^

The SSI-R is a self-report questionnaire used to measure sexual shame. The SSI-R consists of the three subscales (1) sexual inferiority (3 items), (2) relational sexual shame (4 items), and (3) internalized sexual shame (3 items). An example of sexual inferiority is ‘I worry about being able to sexually satisfy my partner’, an example of relational sexual shame is ‘I am afraid of sharing my private sexual thoughts with my partner’, and an example of internalized sexual shame is ‘I replay sexual experiences I am ashamed of over and over in my mind’. All items were scored on a 6-point Likert-scale ranging from strongly disagree to strongly agree. The subscales were added together to create the total sexual shame scale score, with higher scores indicating higher levels of sexual shame (ranging from 10 to 60). Cronbach’s alpha has been shown to range from 0.76 to 0.82 for the SSI-R, indicating a good level of internal consistency^5^.

### Statistical analysis and power

Statistical analyses were performed using IBM SPSS Statistics version 27 and IBM SPSS Amos version 26 (SPSS Inc., Chicago, IL, USA). A path analysis model was used to evaluate the relationship between sexual shame, expressive suppression, cognitive reappraisal and sexual desire. Sexual shame, expressive suppression, and cognitive reappraisal were modeled as exogenous variables. Structural pathways were specified to sexual desire, which was modeled as an endogenous variable. Multiple regression analysis and multivariate analysis of variance (MANOVA) was used to investigate potential relationships between emotion regulation, sexual desire, and sexual shame and to investigate gender differences. Prior to analyses, assumptions of normality and homogeneity of variance were evaluated. The assumption of homogeneity of variance-covariances matrices was shown to be violated (p < 0.001), Pillai’s Trace was therefore used instead of Wilk’s Lambda as it is a more robust test statistic for non-normal and unbalanced samples^28^. A power calculation revealed that the sample size had a statistical power > 95% to detect medium effect sizes (d = 0.50) with α = 0.05, calculated with GPower version 3.1.9.7.

### Ethics statement

The study was reviewed and approved by the local ethics committee at Inland Norway University of Applied Sciences (LEFK 21/01894) and conducted in accordance with the Declaration of Helsinki (World Medical Association, 2011). The participants provided written informed consent prior to participating in this study.

## Results

### Effects of expressive suppression, cognitive reappraisal, and sexual shame on sexual desire

Participants’ mean scores on *ERQ*, *SDI-2*, and *SSI-R* are depicted in Table 1. Table 2 describes the univariate correlations between the SDI-2, ERQ-10, and SSI-R.

**Table 1:** Summary of means and standard deviations for scores on the SDI-2, ERQ-10, and SSI-R.

	*n*	*M*	*SD*
Sexual desire (SDI-2) [range 0–101]	218	52.09	13.47
Partner-focused SD [range 0–54]	218	26.12	7.29
General dyadic SD [range 0–16]	218	7.61	3.63
Solitary SD [range 0–31]	218	18.35	6.37
Emotion regulation (ERQ-10)			
Expressive suppression [range 4–28]	218	13.17	4.85
Cognitive reappraisal [range 6–42]	218	28.76	5.82
Sexual shame (SSI-R) [range 10–60]	218	23.33	8.94

*SDI-2* sexual desire inventory-2, *ERQ-10* emotion regulation questionnaire, *SSIR* sexual same inventory—revised.

**Table 2:** Univariate correlations between the SDI-2, ERQ-10, and SSI-R.

	1	2	3	4	5	6	7
1. Sexual desire (total score)	–						
2. Partner-focused SD	0.82**	–					
3. General dyadic SD	0.68**	0.37**	–				
4. Solitary SD	0.79**	0.37**	0.44**	–			
5. Expressive suppression	0.02	− 0.03	0.07	0.04	–		
6. Cognitive reappraisal	0.17*	0.21**	0.13	0.04	− 0.05	–	
7. Sexual shame	0.03	− 0.13	0.12	0.14*	0.25**	− 0.13*	–

**p < 0.01; *p < 0.05.

**Figure 1:**
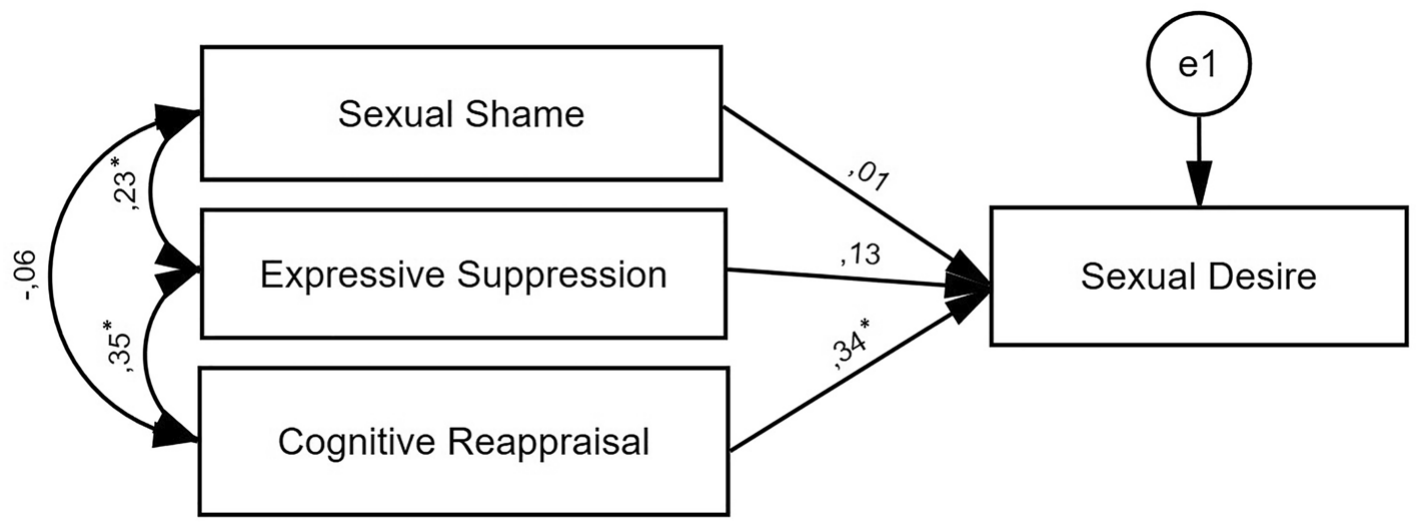
Standardized structural path estimates in a model testing the relationship between sexual shame, expressive suppression, and cognitive reappraisal (standardized regression coefficients and correlation between variables). *p < 0.001.

Standardized path estimates from the model are shown in Fig. 1. Fitness indices showed that the conceptual model of the study had a good fitness. The model indicated that cognitive reappraisal was significantly related to sexual desire (*β* = 0.34, p < 0.001), whereas sexual shame and expressive suppression were unrelated to sexual desire.

A multiple regression analysis was run to investigate whether cognitive reappraisal, emotional suppression, and sexual shame could predict sexual desire. Results revealed that the model accounted for 15,6% of variation in participants sexual desire scores, *R*^2^ = 0.16, F(3, 214) = 14.385, *p* < *0.0*01 and confirmed that, of the three independent variables, only cognitive reappraisal predicted sexual desire, β = 0.343, *t*(218) = 5.09, *p* < 001, CI [0.407, 0.920]. Further investigation of the subscales of the SDI-2 showed that cognitive reappraisal predicted partner-focused sexual desire, F(1, 214) = 8.86, p = 0.003, partial η^2^ = 0.04, and general dyadic sexual desire for an attractive person, F(1, 214) = 4.54, p = 0.03, partial η^2^ = 0.02, but not solitary sexual desire, p > 0.05. Expressive suppression was not found to predict any of the subscales of the SDI-2, all p > 0.05. Sexual shame was found to predict solitary sexual desire, F(1, 214) = 4.22, p = 0.04, partial η^2^ = 0.02, but not partner-focused sexual desire or general dyadic sexual desire for an attractive person, both p < 0.05.

### Gender differences

A MANOVA was run to investigate gender differences on sexual desire, expressive suppression, sexual shame and cognitive reappraisal (see Table 3). A significant main effect of gender was found, Pillai’s Trace = 0.223, F(8, 209) = 7.51, p < 0.001, partial η^2^ = 0.223. Separate univariate tests revealed that gender only had a significant effect on expressive suppression, with men scoring higher (*M* = 16.16) compared to women (*M* = 13.38), F(1, 216) = 24.968, p < 0.001; partial η^2^ = 0.104. No significant effects of gender on sexual desire, sexual shame, or cognitive reappraisal were found, all. p > 0.05. In addition, correlations between sexual desire and expressive suppression, cognitive reappraisal, and sexual shame were investigated separately for each gender. For women, sexual desire was significantly correlated with cognitive reappraisal, r(149) = 0.20, p = 0.01, but not with expressive suppression or sexual shame, all p > 0.05. For men, sexual desire was not correlated with cognitive reappraisal, expressive suppression, or sexual shame, all p > 0.05.

**Table 3:** Gender differences in expressive suppression, cognitive reappraisal, and sexual shame on sexual desire.

	Females (n = 151)	Males (n = 67)			
	M (SD)	M (SD)	F	*p*	Partial η^2^
Sexual desire	47.62 (10.58)	50.01 (8.32)	2.70	0.102	0.012
Emotion regulation					
Expressive suppression	13.38 (3.57)	16.16 (4.24)	24.97	< 0.001*	0.104
Cognitive reappraisal	27.61 (4.99)	28.19 (5.54)	0.59	0.442	0.003
Sexual shame	23.29 (9.63)	23.42 (7.21)	0.009	0.923	< 0.001

SD standard deviation.

*p < 0.001.

## Discussion

The current study aimed to investigate the effects of emotion regulation strategies, sexual shame, and gender on sexual desire. Results showed that cognitive reappraisal, but not expressive suppression or sexual shame predicted participants’ sexual desire. More specifically, it was found that cognitive reappraisal predicted sexual desire for others but not solitary sexual desire suggesting that cognitive reappraisal is mainly relevant for the relational focus of sexual desire and not sexual desire per se. No gender differences were found for sexual desire, sexual shame, or cognitive reappraisal. However, men did score higher on expressive suppression compared to women. Also, while cognitive reappraisal was related to sexual desire in women, no such relationship was found in men.

The finding that sexual shame did not have a negative effect on sexual desire contradicted our initial expectation and suggests that sexual shame may be less of a factor in sexual desire than previously assumed^5,13^. Instead, results support the idea that it is possible to experience sexual desire and sexual shame in tandem and that the experience of shame does not necessarily affect the experience of sexual desire. However, viewing sexual shame and desire as a linear relationship may impair the potential alternative explanations that might exist, and could be an interesting venue to examine in future research.

The prediction that expressive suppression would have a negative effect on sexual desire was also not supported. This was rather unanticipated since previous research has found a relationship between sexual desire and sexual arousal regulation^29^. However, while this previous study investigated sexual arousal regulation the current study focused on general emotion regulation, which could explain the discrepancy in results. Also, since expressive suppression is response-focused and takes place after the emotion has already been generated, while sexual desire can be present without the drive to want to engage in sexual behavior, sexual desire may arise before emotions are generated^30^. This supports the idea that sexual desire may not be affected by expressive suppression.

The positive effect of cognitive reappraisal on sexual desire can be explained by looking at the motivational perspective of how desire is generally regulated. Since sexual desire lies on a spectrum of motivation affected by urges and desires to achieve pleasure and prevent the unpleasant, behavior to seek the pleasurable can be linked to how we regulate emotions in order to achieve these pleasurable states^31^. In other words, the regulation of emotions through antecedent-focused strategies (e.g. before the emotion- generating process is completed) will affect cognition and emotion through the appraisal of what is deemed desirable and thus, regulates approach- or avoidance behavior towards the object of desire, also in the sexual realm^31,32^. In sum, this implies that the better people are at using cognitive reappraisal to regulate emotions, the more they are able to upregulate the positive aspects related to sexual desire^33^.

Study results replicate previous findings showing that men engage in the suppression of emotional expressivity to a larger degree compared to women. Potential reasons why men rely more on this response-focused emotion regulation strategy could be related to gender roles, societal expectations, and the various environmental systems men go through in their development^22,34^. However, counter to previous findings, no gender differences were found for cognitive reappraisal. One potential explanation for this finding is that the participants in the current study were mostly young students, attending higher education. Potentially, the men in this specific demographic group may engage more in cognitive reappraisal strategies compared to men in the general population.

Interestingly, no gender differences were found on measures of sexual shame or sexual desire. Earlier research has commonly found that women experience more stigmatization related to sexual behavior and sexual emotions compared to men^35,36^. These findings suggest that we might be in the midst of a cultural shift where women’s sexuality is less stigmatized and more openly discussed. In social media, elementary education, and in the news, the topics sexual health, sexuality, and sexual behavior are increasingly receiving more awareness. Viewing earlier research on sexual desire chronologically, it can be seen that gender differences in sexual desire have been decreasing during the last two decades^4,6–8^.

The current study has several limitations. Since the sample was largely recruited through the use of social media and facebook-groups, recruitment was subject to convenience sampling. There was a large proportion of women (69%) participating, and most participants were young (80.6% between the ages 18–29). Consequently, results cannot be generalized to older populations or populations outside Norway. Future studies with larger sample sizes and from other populations are suggested to verify the results and to get a better understanding of generational differences. Another limitation to the study was that the majority of participants were psychology students, a rather homogenous group, which could be a source of bias and an explanation for why few gender differences were found on the administered measures.

To summarize, the current study did not find gender differences in sexual shame, sexual desire, and cognitive reappraisal, which has been found previously in older studies. However, the use of cognitive reappraisal in everyday life was found to be associated with increased sexual desire, at least in women. Results from the current study indicates that the inclination toward cognitive reappraisal as a preferred emotion regulation strategy may positively affect the strength of sexual desire.

## Data Availability

The raw data supporting the conclusions of this article can be made available on request to the corresponding author (carolien.konijnenberg@inn.no).
